# Supplementary material for: Overexpression of Saussurea involucrata dehydrin gene SiDHN promotes cold and drought tolerance in transgenic tomato plants
Source: PLoS One. 2019 Nov 18;14(11):e0225090. doi: 10.1371/journal.pone.0225090 (PMC6860438; doi:10.1371/journal.pone.0225090)
Supplement: Attachment — Submitted filename: Response to Reviewers.docx [file pone.0225090.s001.docx]

September 10, 2019

T. R. Ganapathi, Ph.D.

Academic Editor

*PLOS ONE*

Dear editor:

Thank you very much for reviewing our manuscript and giving us the opportunity to submit a revised draft of our manuscript titled “Overexpression of *Saussurea involucrata* Dehydrin Gene *SiDHN* Promotes Cold and Drought Tolerance in Transgenic Tomato Plants” to *PLOS ONE*. We greatly appreciate the time and effort that you and the reviewers have dedicated to providing your valuable feedback on our manuscript. We are also grateful to the reviewers for their insightful comments on our paper.

We have used a plagiarism checker available to us to ensure that there is no occurrence of overlapping text. We have also incorporated changes to reflect most of the suggestions provided by the reviewers, which can be found in the file labelled ‘Revised Manuscript with Track Changes’.

Please also find below a point-by-point response to the reviewer’s comments and concerns, The reviewers’ comments are italicized and our responses are in plain text. [Please note that the Track Changes function has caused the line numbers to change; as a result, the line numbers in the file with Track Changes are different from those in the file ‘without Track Changes’. The line numbers shown in the response are correspondent to those in the file without Track Changes (Manuscript)].

**Comments from Reviewer 1**

**Comment 1:** *Abstract: The term “tolerance” appears to be more appropriate instead of “resistance” for improved performances against abiotic stresses. Conclusion need to be written more specifically.*

**Response:** Thank you for pointing this out, and we agree that these should be changed. Accordingly, we have changed the term and improved the conclusion in the abstract. Please refer to lines 38 and 50 – 55.

**Comment 2:** *Introduction: Statements need to be supported with published scientific literature. For example please refer line no 92-95. Necessity of the research work also needs to be clearly stated.*

**Response:** Thank you very much for pointing out. We have ensured that all statements are supported by the published literature. We have also edited and rearranged the statements so that the necessity of the research work is more clearly stated, which can be found in lines 82 – 95.

**Comment 3:** *Materials and methods: Plant lets of S. involucrate were regenerated through tissue culture, therefore use term “plantlets” instead of “seedlings”. Details are missing on explants, medium etc. for tissue culture of the plant. The purpose of using tissue culture raised plants need to be mentioned in the manuscript.*

**Response:** We agree with the reviewer on these points. Accordingly, we have changed from ‘seedlings’ to ‘plantlets’, where we find necessary, and have provided the details on explants, medium, purpose of using tissues culture to raise plants. We have also included other details necessary for the understanding of the section. We invite the reviewer to check the ‘Plant materials and growth conditions’ section in lines 111 – 122.

**Comment 4:** *Materials and methods: Sequence analysis of SiDHN: Details on primer designing and sequencing of the PCR amplicon are missing.*

**Response:** We agree with you on this point. We have incorporated the details on how the primer was designed at the bottom of Table 1, as well as on the sequencing of PCR amplicon, which was done after plasmid extraction (Lines 210 – 220).

**Comment 5:** *Materials and methods: Plant Transformation: Details are missing on cloning of the gene and Agrobacterium transformation. Important details are missing on genetic transformation of tomato such as explant, co-cultivation, regeneration etc. Details are missing on RNA isolation, RT etc.*

**Response:** Thank you so much for pointing this out. We agree with you and have incorporated new details on cloning and Agrobacterium transformation, as well as of transformation of tomato. We invite you to check the ‘Plant transformation and transgenic tomato identification’ section (Lines 210 – 237), which has been largely edited.

**Comment 6:** *Materials and methods: Stress tolerance: How many plants were used for treatment? The stress treatment for short period of 2h imposes shock to the plants. Why longer treatments were not given to assess the stress tolerance and real response of the plants? Details pertaining to the bio-chemical analyses are missing. It is important to mention the biological and technical replicates for the analyses.*

**Response:** Thank you for this comment. Please find a point-by-point response below.

**Response to** *“How many plants were used for treatment?”*: We used 3 wild-type and 9 T_2_ transgenic tomato plants, both were ten weeks old. We have also added the corresponding details in the manuscript. Please refer to lines 141 – 142.

**Response to** *“The stress treatment for short period of 2h imposes shock to the plants”*: We apologize for this error. The stress treatment was, in fact, carried out for 48 h (or 2 d), and we have changed the content accordingly. Please see line 143.

**Response to** *“Why longer treatments were not given to assess the stress tolerance and real response of the plants?”*: Thank you for pointing this out. We did, in fact, carry out the treatment at a longer period, at which the stress tolerance appeared to be deteriorated. We decided to not include the data in the manuscript; however, we have included some details in the manuscript (results section) to indicate the finding. Please refer to lines 396 – 397.

**Response to** *“Details pertaining to the bio-chemical analyses are missing”*: We apologize for this unclarity. We have included more details pertaining the biochemical analyses, which can be found at the beginning of each paragraph of the sections ‘Measurement of chlorophyll pigment content’ (lines 240 – 253) and ‘Determination of morphological and physiological traits’ (lines 268 – 270, lines 276 – 277, lines 283 – 285, lines 293 – 297, lines 304 – 306).

**Response to** *“It is important to mention the biological and technical replicates for the analyses”*: We have included sentences stating that “Each experiment was carried out in triplicate, and three replicate samples were prepared in parallel” in all sections, where we find appropriate, such as in lines 323 – 324.

**Comment 7:** *Results: Transgenic confirmation with mere kanamycin selection and PCR may not be sufficient. Statistical significance, if any, for the parameters among the transgenic and wild types need to be clearly stated rather than just mentioning the higher or lower change. The reason behind studying the transcript expression of few genes is not clearly stated. What was the criterion for selection of the genes? Whether, the PCR amplicons for the selected genes were confirmed by sequencing? What was the relation between the transcript level and activity of the coded anti-oxidant enzymes?*

**Response:** Thank you for pointing this out. We carried out the study following a number of publications that have used the same selection method (e.g., reference [22]), which have been proven to be sufficient. We have edited the sentences, incorporating the term ‘significantly different’ or ‘significantly higher or lower’ in various areas of the manuscript, where we find appropriate. We have also largely modified the content to address the concerns above, but made throughout different areas on the manuscript. We invite the reviewer to recheck out new content, such as lines 578 – 582, lines 548 – 556, and the discussion section lines 674 – 706.

**Comment 8:** *Discussion: Author stated that “expression of SiDHN was induced in S. involucrata by cold temperatures and drought (Fig 2), confirming that the gene participates in the plant’s defenses to abiotic stress”. However, it is necessary to understand that mere change in expression level of any gene does not confirm its role in plant defence, functional validation is required in support of such statement.*

**Response:** Thank you for this comment. In fact, the participation of *SiDHN* in the plant’s defenses to abiotic stress has been reported previously, as can be found in, for example, reference [22] and [57], and our study confirmed such participation.

**Comments from Reviewer 2**

**Comment 1:** *Introduction, material and methods and results are well covered, but the discussion is poorly written. They must explain/discuss the findings in the light of available literature.*

**Response:** Thank you for pointing this out. We have largely edited and rearranged the entire discussion section so that it is presented in the way that the reviewer suggested. We do hope that it is sufficient. We invite you to check the ‘Discussion’ section in lines 609 – 716.

**Comment 2:** *Resolution of most of the figures is poor.*

**Response:** Thank you for pointing this out. We have increased the resolution of the figures, which we find most important for the comparison.

**Comment 3:** *Manuscript requires extensive editing (scientific as well as grammatical). There are several errors across MS. A few are:*

*Line 45: Use specific word than slightly.*

*Line 50: within the transgenic?*

*Line 73: tied to the acquisition?*

*Line 73: tied to the acquisition?*

*Line 192: change 13000 rpm to x g*

*Youngest fully expanded leaves?*

*Line 120-121: gene and scientific name in italics*

*Line 193: Supernatant fluid?*

**Response:** We appreciate the reviewer for pointing this out. We have corrected/changed the errors as recommended in the comment, as well as in the entire manuscript as we spotted them. Please refer the specific response below.

**Response to** *“Line 45: Use specific word than slightly”:* We have changed the sentence to avoid using the word ‘slightly’ in this sentence (lines 45 – 46), as well as in all other sentences, where applicable. In many cases, we have instead incorporated the term ‘significantly’ (based on the statistical analysis).

**Response to** *“Line 50: within the transgenic?”*: We have changed the expression to ‘in the transgenic plants’ (lines 46 – 47). We have also used similar expression throughout the manuscript, where we find appropriate.

**Response to** *“Line 73: tied to the acquisition?”*: We have changed this expression to ‘are closely linked with’. Please refer to line 73.

**Response to** *“Line 192: change 13000 rpm to x g”*: We have changed rpm to xg throughout the manuscript. The original line 192 has now been moved to a new line 332.

**Response to** *“Youngest fully expanded leaves?”*: The intent of this phrase is to indicate that the leaves are young but are fully-grown. Thus, we have changed this to ‘young, fully-grown leaves’. Please refer to lines 287 – 288.

**Response to** *“Line 120-121: gene and scientific name in italics”*: We have italicized the gene and scientific names accordingly throughout the manuscript.

**Response to** *“Line 193: Supernatant fluid?”:* We have changed this to ‘the supernatant’, and have also rearranged the sentence. Please refer to line 309.

**Comment 4:** *Line 40-45: Write shorter and clear sentences.*

**Response:** We have removed repetition and redundancy in these sentences and changed them to:

‘We observed that in response to stresses, the *SiDHN* transgenic tomato plants had increased contents of chlorophyll a and b, carotenoid and relative water content compared with wild-type plants. They also had higher maximal photochemical efficiency of photosystem II and accumulated more proline and soluble sugar. Compared to those wild-type plants, malondialdehyde content and relative electron leakage in transgenic plants were not significantly increased, and H_2_O_2_ and O_2_^-^ contents in transgenic tomato plants were significantly decreased.’

Please refer to lines 40 – 47.

**Comment 5:** *Line 83-84: References*

**Response:** Thank you for pointing this out. We have recounted the references and have inserted the appropriate reference numbers accordingly. Please refer to lines 82 – 84.

**Comment 6:** *Line 137: Whether author are sure they have used just 80µg/L kanamycin for selection?*

**Response:** Thank you for this question. We carried out the experiment according to the previously published article in which 80µg/L kanamycin was used for selection. We also carried out RT-PCR to confirm that the gene has been transformed.

**Comment 7:** *For the freezing stress, authors gave treatment for 4⁰C/2h and 0⁰C/2h. Why longer duration not selected?*

**Response:** We apologize for this mistake. We, in fact, conducted the treatment at each temperature for 48 h and have accordingly corrected the detail. We also carried out the treatments at a longer time period, but decided not to include it in the data. We have, however, stated in the manuscript (result section) that the tolerance was decreased at a period longer than 48 h, which can be found in lines 395 – 397.

**Comment 8:** *Why two methods were used for CAT & POX estimation?*

**Response:** We cited two references (for each estimation) that describe the same method. We have reduced the number of reference and cited to the one published in earlier year. Please refer to lines 336 and 339.

**Comment 9:** *What is the relevance of ref. 35?*

**Response:** This appears to be a mistake on our end, and we apologize for that. We, however, have made extensive changes in the manuscript, whereby reference [35] is now no longer relevant. We invite you to check the edited content, especially for the section ‘Plant treatments’ (lines 132 – 151), in which the reference [35] was originally cited.

**Comment 10:** *Conditions for the enzyme analysis (4⁰C/2h and 0⁰C/2h) and gene expression analysis (4°C chamber for 48 h and then kept at 0°C for 48 h) were different. Why?*

**Response:** We apologize for the error. This should, in fact, be 2 d or 48 h. However, I have changed it to 48 h for consistency.

**Comment 11:** *Line 366: Authors claim that over-expression of SiDHN does not have any visible effect on transgenic plants. It is contradictory to what they mentioned in 326-355. They must explain why transgenic plants have higher chlorophyll content and a shorter height than control plants? WT-plants does not appear healthy. Under normal growth conditions, why leaves will have less chlorophyll. Why will dehydrin give advantage to transgenic plants under normal condition? They must explain this.*

**Response:** We apologize for this mistake. The overexpression of *SiDHN* did, in fact, affect the phenotypes of the transgenic plants. Thus, we have made some revisions regarding this issue both in the results and the discussion sections, which should cover the questions stated above. We invite the reviewer to re-check the revised contents; for example, in the ‘results’ sections lines 470 – 471, and in the ‘discussion’ section lines 632 – 648.

**Comment 12:** *213-214: Dehydrated on filter paper: Is this a standard method for drought stress? To me, it’s not convincing as there can be many variables.*

**Response:** Thank you for this comment. We, in fact, carried out this experiment following the method described in Zhu et al [23], which has been proven to be sufficient.

**Comment 13:** *235: For real-time PCR, whether the author followed the MIQE guidelines? If yes, the same should be mentioned.*

**Response:** Thank you for pointing this out. Yes, we did follow the MIQE guidelines and accordingly, have included the statement in the manuscript. Please refer to line 161.

**Comment 14:** *Whether MEANS in Fig. 2 were compared (statistics)? Same is not reflected.*

**Response:** Thank you for this comment. We did all comparisons statistically and have included the details in the description of figure 2, as well as others where we find necessary for the better understanding of the content.

**Comment 15:** *There was a continuous increase in SiDHN expression under cold stress during 0-48h, unlike in drought. Why was the expression of SiDHN not monitored beyond 48h?*

**Response:** We, in fact, carried out the treatment at each temperature beyond 48 h but decided not to include it in the data. We have, however, stated the finding in the manuscript (result section), which can be found in lines 396 – 397.

**Comment 16:** *316-319: SiDHN from S. involucrata was over-expressed (heterologous) in tomato plants. So there should not be any expression of the gene in WT-plants. But in Fig. 3A, a certain level of expression is shown. Also, they should explain if there is no expression in WT-plants, how 292,241, 139-fold mRNA levels than WT-plants was observed?*

**Response:** Thank you for this comment. Yes, there was indeed no expression of the *SiDHN* gene in the wild-type potato plants. The comparison was based on the assumption set in ‘Statistical analysis’ section, which states that ‘the expression level at 0 h (control time point) was defined as 1.0’. However, we decided to not include these numbers in the manuscript, rather we stated that ‘Three independent homozygous lines (OE-2, OE-5, and OE-8) that had higher expression levels of *SiDHN* were selected for further studies (Fig 3).’ Please refer to lines 422 – 423.

**Comment 17:** *They have not included vector-control plants for any of their studies. Why?*

**Response:** Thank you for pointing this out. We carried out the experiment following the previous publication on *SiDHN* (e.g. reference [22]); thus, we decided that not including vector-control plants should be appropriate. And we believe that the results should provide insights into the understanding of *SiDHN* gene expression to some extent.

**Comment 18:** *Fig. 7: Looking at fig, it's difficult to believe uniformity among control and transgenic plants. To me, a lot of variation is visible even on day 0 in plant phenotype between even cold/drought stress experiments.*

**Response:** We agree with the reviewer on this and have changed/edited some sentences so that the content more clearly presents such differences. We invite the reviewer to re-check the section ‘Morphological changes of *SiDHN*-overexpressing transgenic tomato plants under cold and drought stress’ (Lines 470 – 471).

**Comment 19:** *454-455: Fig. 10E, F & 11 E,F should be 9 EF & 12 E,F.*

**Response:** Thank you very much for pointing out this error. We have changed the content accordingly. Please see lines 558 – 559.

**Comment 20:** *Why levels of H2O2 and O2- were not monitored on 0, 24, 48h. That would have been one of the important aspects to link with the status of downstream antioxidant enzymes. Levels of H2O2 and O2 should be included in duration dependent ie. 0, 24, 48h to correlate the things with levels of enzymes.*

**Response:** We decided to determine the levels of *H_2_O_2_ and O^2-^* in correspondent to the enzyme activity (Fig. 9), which was determined at the end of the treatment (48 h). We think that in our future study, we should determine such levels at various times as the reviewer suggested. In addition, because the gene expression does not necessarily determine the enzyme activity; therefore, we measured the levels of the levels of *H_2_O_2_ and O^2-^* only when the enzyme activity was measured so that we can better correlate the results.

**Comment 21:** *Line 524: How would the author explain the higher levels of expression of SiDHN in leaves?*

**Response:** There have been only a few studies on the effects of *SiDHN* overexpression on plant growth and development. For example, Nylander et al. [55] believed the expression of the dehydrin gene may be related to its function and can also be affected by different stages of plant development under stress. Stupnikova et al. [56] proposed that plants have different resilience at different developmental stages: the expression of dehydrin is high when they are under high resilience. These studies have also shown that the expressions of many dehydrins are specific to tissues, and the growth conditions can also affects the expression levels. The studies, however, have not focused on the expression in different plant tissues. Nonetheless, based on out results, we believe that the higher level of expression in leaves may be due to high resilience in leaves, which may be the first target of stress. We have accordingly incorporated the mention in lines 625 – 628.

**Comment 22:** *Fig: 7, whether the recovery of plants was observed for a longer duration. What were the results post 3days recovery?*

**Response:** Thank you for this comment. We did indeed observed the recovery after 3 days (we decided to not include the data) and found that the wild-type tomato plants could not recover and showed no signs of growth, while the transgenic tomato plants could recover (side buds were exposed in the stem, and middle and bottom leaves were restored) only when the apex remained intact.

**Comment 23:** *Levels of SiDHN was found highest in OE8, but levels of most of the antioxidant enzymes including tolerance and survival under stress were observed in OE2 (Recovery). How would the author explain it? Similarly, in the case of expression of analysis of APX, SOD, POX (Fig. 10) shows an increase in their levels with duration. But it’s difficult to understand unless it is shown that H2O2/O2- levels also increased.*

**Response:** Thank you very much for this question. In fact, the expression level of *SiDHN* was highest in OE-2 (was lowest in OE-8; as can be seen in Fig 3) and is correspondent with its tolerance and survival under stress (e.g. Fig 7). Regarding the expression of APX, SOD and POX, we invite the reviewer to compare the levels of APX, SOD and POX at 48 h in Fig 10 with the levels of H_2_O_2_ and O^2-^ (measured at 48 h) in Fig 9, particularly for OE-2: we can see that the levels of APX, SOD and POX were highest, while the levels of H_2_O_2_ and O^2-^ were lowest. We do hope that the explanation is clear.

In addition to the corrections above, we have also checked and corrected other errors found during the revision.

We hope that you find our responses and corrections satisfactory. We look forward to hearing from you regarding our submission and are pleased to respond to any further questions and comments you may have.

Sincerely,

Jianbo Zhu

Key Laboratory of Agricultural Biotechnology

College of Life Science, Shihezi University,

Shihezi, Xinjiang, China

Phone No: 18935701090

Fax No: 18935701090

E-mail Address: jianboz9@sina.com
