# Supplementary material for: Overexpression of Saussurea involucrata dehydrin gene SiDHN promotes cold and drought tolerance in transgenic tomato plants
Source: PLoS One. 2019 Nov 18;14(11):e0225090. doi: 10.1371/journal.pone.0225090 (PMC6860438; doi:10.1371/journal.pone.0225090)
Supplement: Attachment — Submitted filename: Response to Reviewers.docx [file pone.0225090.s002.docx]

October 24, 2019

T. R. Ganapathi, Ph.D.

Academic Editor

*PLOS ONE*

Dear editor:

We highly appreciate you and the reviewers for reviewing our manuscript and giving us the opportunity to submit a revised draft of our manuscript titled “Overexpression of *Saussurea involucrata* Dehydrin Gene *SiDHN* Promotes Cold and Drought Tolerance in Transgenic Tomato Plants” to *PLOS ONE*. We also appreciate your and the reviewers’ valuable and insightful feedback on our manuscript.

We have carefully considered the reviewer’s comments and respond to all of the suggested revisions in more detail below. The reviewers’ comments are italics and our responses are in plain text; line and page numbers refer to the revised manuscript.

**Comments from Reviewer 1**

**Comment 1:** *Abstract: The term “tolerance” appears to be more appropriate instead of “resistance” for improved performances against abiotic stresses. Please check line no 54-56.*

**Response:** Thank you for this comment. We agree to the reviewer and have changed the term accordingly. Please see line 56.

**Comment 2:** *Materials and methods:*

**Comment:** *Line 111: Plantlets of S. involucrata were prepared by tissue culture. It is not clear why authors followed tissue culture to produce plantlets. Moreover, progeny obtained through indirect regeneration may not be true to type due to somaclonal variations. Whether the obtained plantlets were not hardened?*

**Response:** Thank you for this comment. We followed the tissue culture to produce plantlets due to the following reasons:

1. Due to its special growth conditions, artificial propagation of *Saussurea involucrata* is very difficult. Because plant tissue culture technology is not limited by time, place, climate and other environmental conditions, the culture conditions can be easily controlled and can achieve long-term preservation. This not only allows researchers to produce the plants at a large scale at any time, but also provides an important way for mass propagation of medicinal plant *S. involucrata*.
2. The sequence of *SiDHN* was obtained by sequencing of the full-length cDNA library of *Saussurea involucrata*. And the materials used to build the library were obtained by tissue culture seedling that has been preserved in our laboratory for a long period of time. More importantly, the cloning of *SiDHN* used in this study was from the same tissue culture and materials.
3. We compared the cDNA sequence of *SiDHN* gene cloned in this paper with the full-length cDNA sequence of *Saussurea involucrata* from the database, and found that they were 100% identical. This should be an indication that our method is reliable.
4. This approach has been widely reported. Please see below for example publications that have conducted similar experiments and have used similar methods.

- [Xinyong Guo,](https://www.sciencedirect.com/science/article/pii/S0168945218316029#!) [Li Zhang](https://www.sciencedirect.com/science/article/pii/S0168945218316029#!), [Gaoquan Dong](https://www.sciencedirect.com/science/article/pii/S0168945218316029#!), [Zhihua Xu](https://www.sciencedirect.com/science/article/pii/S0168945218316029#!), [Guiming Li](https://www.sciencedirect.com/science/article/pii/S0168945218316029#!), [Ning Liu](https://www.sciencedirect.com/science/article/pii/S0168945218316029#!), [Aiying Wang](https://www.sciencedirect.com/science/article/pii/S0168945218316029#!), [Jianbo Zhu](https://www.sciencedirect.com/science/article/pii/S0168945218316029#!). A novel cold-regulated protein isolated from *Saussurea involucrata* confers cold and drought tolerance in transgenic tobacco (*Nicotiana tabacum*). Plant Science, 2019, 289: 110246.
- Linhua Zhang, Li Sun, Li Zhang, Hongling Qiu, Chao Liu, Aiying Wang, Jianbo Zhu. A Cu/Zn superoxide dismutase gene from *Saussurea involucrata Kar*. & Kir., *SiCSD*, enhances drought, cold, and oxidative stress in transgenic tobacco, Canadian Journal of Plant Science. 2017, 97(5): 816-826.
- Liu HL, Shen HT, Chen C, Zhou XR, Liu H, and Zhu JB. Identification of a putative stearoyl acyl-carrier-protein desaturase gene from *Saussurea involucrata.* Biologia Plantarum. 2015, 59 (2): 316-324.

**Comment:** *Line No 117: NAA (N-acetyl-aspartate); Please check full form of NAA.*

**Response:** Thank you for pointing this out. We have changed it to the correct name, which is ‘1-Naphthaleneacetic acid’. Please see line 117.

**Comment:** *Lines 123-124: Tomato plant variety ‘Yaxin 87-5’ used as transgenic plants were grown from 124 seeds provided by Yaxin Seed Co. Ltd. (Shihezhi City, Xinjiang, China). Please rewrite the sentence, as meaning is not clear. Whether, genetic transformation of tomato was carried out by the authors or transgenic seeds provided by Yaxin Seed Co. Ltd were used to grow the transgenic plants for the present study?*

**Response:** Thank you for this comment. Yaxin Seed Co. Ltd. provided the wild-type tomato plant seeds (variety "Yaxin 87-5"), not the transgenic tomato plants/seeds. We then grew tomato plants from the seeds (denoted as wild-type tomato plants), and the plants obtained were used to generate the transgenic tomato plants (denoted as transgenic tomato plants).

Accordingly, we have changed the sentence to the following: ‘Seeds of tomato plant variety ‘Yaxin 87-5’ (wild-type) were provided by Yaxin Seed Co. Ltd. (Shihezhi City, Xinjiang, China). The wild-type tomato plants were grown from the seeds in our laboratory and were used to produce transgenic tomato plants.’ Please refer to lines 123-126.

We do hope that it is now clearer.

**Comment:** *Line 126-127: After that, they were sown in plastic pots ....Do you mean the plantlets were transplanted in plastic pots....*

**Response:** Thank you for the comment. That is correct. Accordingly, we have changed the sentence to ‘After that, the plantlets were transplanted in plastic pots.’ line 128.

**Comment:** *Line 146-148: Top-second and -third leaves of the plants were harvested at 4°C and 0 °C after the 48 h treatment and at 0, 16 and 24 d after the 148 drought treatment. Meaning of the sentence is not clear.*

**Response:** Thank you for this comment. Please note that there should be no ‘148’ in the manuscript; we are not sure if this number shows in the reviewer’s file.

We collected the second and the third leaves from the top of the plants. The leaves of the cold-treated plants (at both temperatures) were collected at 48 h, which is the end of the treatment, and the leaves of the drought-treated plants were collected at day 0, 16, and 24.

We have changed the sentence to: ‘Tomato leaves (the second and third leaves from the top) were collected after 48 h of the cold treatments (4^o^C and 0^o^C), or after 0, 16 and 24 d of the drought treatment.’ We do hope that it is now clearer. Please see lines 148-150.

**Comment:** *Line 217-218: ....and the obtained plasmid was regenerated in Escherichia coli DH5 alpha. Do you mean “the obtained plasmid was transformed in Escherichia coli strain DH5α ?”*

**Response:** Thank you for this comment. Yes, it is in fact more accurate to write ‘… and the obtained plasmid was transformed into *Escherichia coli* strain DH5α’. We have changed the sentence accordingly (lines 218-219).

**Comment:** *Line No. 221-223. To produce transgenic potato plants, the wild-type tomato plants were transformed ….. What do you mean? Transgenic potato plants were developed after transforming tomato?*

**Response:** We apologize for this error and thank you for pointing it out. All should be ‘tomato’, and we have changed all accordingly. We have also ensured that the word ‘potato’ no longer exists in our manuscript. Thank you very much again.

**Comment:** *Lines 258-260: Measurement of chlorophyll pigment content: ....the supernatant was subjected to absorbance measurements at 470 nm, 645 nm, and 663 nm to determine the contents of chlorophyll a (Chl a), chlorophyll b (Chl b), and carotenoids (Car), respectively. Do you mean, the estimations of chl a, chl b and car are based on absorbance at single wavelength?*

**Response:** Thank you for this question. We determined the contents of Chl a, Chl b, and Car using the wavelengths described in the sentence. And the contents were calculated by the following equations:

Chl a = 12.21*A_663_ – 2.81*A_645_

Chl b = 20.13*A_645_ – 5.03*A_663_

Car = (1000*A_470_ – 3.27*Chl a – 104*Chl b)/229.

Accordingly, we have changed the content to ‘After centrifugation, the supernatant was subjected to absorbance measurements at 470 nm, 645 nm, and 663 nm, and the contents of chlorophyll a (Chl a), chlorophyll b (Chl b), and carotenoids (Car) were calculated by the following equations: Chl a = 12.21*A_663_ – 2.81*A_645_; Chl b = 20.13*A_645_ – 5.03*A_663_; and Car = (1000*A_470_ – 3.27*Chl a – 104*Chl b)/229.’ Please refer to lines 262-264.

**Comment:** *Line No 318: .....for 10 min, 200 mL of the supernatant was mixed with 1.8.... Is it 200mL or 200µl?*

**Response:** We appreciate the comment and apologize for the error. This should in fact be 2 mL (not 200 mL). We have corrected the sentence accordingly (line 324).

**Comment 3:** *Results: Author should elaborate the results on effect of cold and drought treatments on physio-biochemical traits in wild type and transgenic plants. Further, relation between the transcript level and activity of the coded anti-oxidant enzymes need to be stated in detail. Fig 5,6: X Axis title: Instead of “Different strains”, “Different lines” is appropriate.*

**Response:** Thank you for this comment. The study showed that overexpression of *SiDHN* alleviated the accumulation of ROS by upregulating the activity of ROS scavenging enzymes SOD, POD, CAT and POX under low temperature and drought stress conditions (Figs. 10 and 11). To study why transgenic plants can maintain relatively high antioxidant enzyme activity under low temperature and drought stress, we measured the expressions of related genes encoding SOD, POD, CAT and APX. RT-PCR results showed that the expressions of *SlSOD*, *SlPOD, SlCAT* and *SlAPX* were significantly up-regulated in *SiDHN* transgenic plants OE-2, OE-5 and OE-8 (Figs. 12 and 13). These results indicate that overexpression of *SiDHN* may be able to improve the expression of antioxidant related genes, thus increasing the activity of the corresponding antioxidant enzymes, enhancing ROS clearance, and in turn reducing cell membrane damage caused by low temperature- and drought-induced oxidative stress.

We have incorporated similar details as those outlined above into the manuscript. We invite the reviewer to check the revised paragraph in pages 17 and 18, and we hope that it is now clearer and sufficient.

Regarding Figs. 5 and 6, we have accordingly changed the X-axis title (from ‘Different strains’ to ‘Different lines’).

**Comment 4:** *Discussion: Line No 651-652:..... in protecting the transgenic potato plants against cold and drought stress. Potato or tomato?*

**Response:** We sincerely apologize for this error and thank you very much for pointing it out. All should be ‘tomato’, and we have changed all accordingly. We have also ensured that the word ‘potato’ no longer exists in our manuscript.

In addition to the corrections above, we have also checked and corrected other errors found during the revision.

We do hope that you find our responses and corrections satisfactory. We look forward to hearing from you regarding our submission and are pleased to respond to any further questions and comments you may have.

Sincerely,

Jianbo Zhu

Key Laboratory of Agricultural Biotechnology

College of Life Science, Shihezi University,

Shihezi, Xinjiang, China

Phone No: 18935701090

Fax No: 18935701090

E-mail Address: jianboz9@sina.com
